# Artemisinin attenuated oxidative stress and apoptosis by inhibiting autophagy in MPP^+^-treated SH-SY5Y cells

**DOI:** 10.1186/s40709-021-00137-6

**Published:** 2021-02-25

**Authors:** Junqiang Yan, Hongxia Ma, Xiaoyi Lai, Jiannan Wu, Anran Liu, Jiarui Huang, Wenjie Sun, Mengmeng Shen, Yude Zhang

**Affiliations:** 1 Molecular Biology Laboratory, The First Affiliated Hospital, College of Clinical Medicine of Henan University of Science and Technology, Jinghua Road 24, Luoyang, Henan 471003 People’s Republic of China; 2 Department of Neurology, The First Affiliated Hospital, College of Clinical Medicine of Henan University of Science and Technology, Luoyang, 471003 People’s Republic of China

**Keywords:** Artemisinin, Parkinson’s disease, Oxidative stress, Apoptosis, Autophagy

## Abstract

**Background:**

Parkinson’s disease (PD) is the second most common neurodegenerative disease after Alzheimer's disease. The oxidative stress is an important component of the pathogenesis of PD. Artemisinin (ART) has antioxidant and neuroprotective effects. The purpose of this study is to explore the neuroprotective effect of ART on 1-methyl-4-phenyliodine iodide (MPP ^+^)-treated SH-SY5Y cells and underlying mechanism.

**Methods:**

We used MPP^+^-treated SH-SY5Y cells to study the neuroprotective effect of ART. Cell viability was measured by MTT assay after incubating the cells with MPP^+^ and/or ART for 24 h. DCFH-DA was used to detect the level of intracellular reactive oxygen species (ROS), and WST-8 was used to detect the level of superoxide dismutase (SOD). The level of intracellular reduced glutathione (GSH) was detected with 5,5΄-dithiobis-(2-nitrobenzoic acid), and the level of malondialdehyde (MDA) was assessed based on the reaction of MDA and thiobarbituric acid. A mitochondrial membrane potential detection kit (JC-1) was used to detect changes in the mitochondrial membrane potential (MMP), and an Annexin V-FITC cell apoptosis kit was used to detect cell apoptosis. The expression levels of caspase-3, cleaved caspase-3 and the autophagy-related proteins LC3, beclin-1, and p62 were detected by Western blotting. In addition, to verify the change in autophagy, we used immunofluorescence to detect the expression of LC3 and p62.

**Results:**

No significant cytotoxicity was observed at ART concentrations up to 40 μM. ART could significantly increase the viability of SH-SY5Y cells treated with MPP^+^ and reduce oxidative stress damage and apoptosis. In addition, the Western blotting and immunofluorescence results showed that MPP^+^ treatment could increase the protein expression of beclin1 and LC3II/LC3I and decrease the protein expression of p62, indicating that MPP^+^ treatment could induce autophagy. Simultaneous treatment with ART and MPP^+^ could decrease the protein expression of beclin1 and LC3II/LC3I and increase the protein expression of p62, indicating that ART could decrease the level of autophagy induced by MPP^+^.

**Conclusion:**

Our results indicate that ART has a protective effect on MPP^+^-treated SH-SY5Y cells by the antioxidant, antiapoptotic activities and inhibition of autophagy. Our findings may provide new hope for the prevention and treatment of PD.

## Background

Parkinson’s disease (PD) is a neurodegenerative disease characterized by the degeneration and death of dopamine (DA) neurons in the substantia nigra of the midbrain [1]. The prevalence rate of PD in China is as high as 1.7% among people above the age of 65 years [2]. The main manifestations of PD include resting tremor, bradykinesia, muscle rigidity and gait disturbance [3]. At present, Parkinson’s disease is mainly treated with drugs that principally relieve symptoms, such as dopamine agonists and L-DOPA. Long-term use of these drugs is often accompanied by a series of adverse reactions [4]. Therefore, exploring the pathogenesis of PD and identifying new drugs that can inhibit damage to DA neurons are of great significance for the treatment of PD.

It has been reported that oxidative stress is an important component of the pathogenesis of PD. Autopsy results of PD patients showed that oxidative stress was present in the substantia nigra of PD patients [5]. Research on the antioxidant system of PD patients showed that the level of reduced glutathione (GSH) in the substantia nigra is significantly reduced, which renders dopamine neurons more sensitive to oxidative stress [6]. In recent years, an increasing number of studies have shown that oxidative stress is the main cause of neuronal apoptosis in PD [7, 8]. Oxidative stress leads to an increase in intracellular reactive oxygen species (ROS). Excessive ROS can oxidize lipids in the neuronal cell membrane, disrupt DA neuron membrane function, or directly damage DNA, ultimately leading to neuronal degeneration. Maruyama et al. [9] found that the level of an endogenous MPTP-like toxin was significantly higher in the cerebrospinal fluid of untreated PD patients than in the cerebrospinal fluid of controls. This toxin can induce dopaminergic SH-SY5Y cell apoptosis, and this effect can be inhibited by antioxidants; this is an indication that ROS may initiate the apoptosis of dopamine neurons. In general, these results directly or indirectly indicate that oxidative stress is involved in the pathogenesis of PD and mediates apoptosis.

Artemisinin (ART), which is extracted from the stems and leaves of *Artemisia annua*, is a well-known antimalarial drug that has saved millions of lives [10, 11]. In recent years, it has been discovered that ART can protect a variety of neurons from oxidative stress damage [12]. Xia Zhao et al. [13] reported that ART decreased the hydrogen peroxide (H_2_O_2_)-induced oxidative damage in SH-SY5Y cells and hippocampal neurons by activating the AMPK pathway. In another study, ART protected rat adrenal pheochromocytoma (PC12) cells and brain primary cortical neurons from sodium nitroprusside-induced oxidative damage through ERK [14]. However, the protective effect of ART on PD has not been studied thus far. Therefore, in this study, we studied the neuroprotective effects of ART in an in vitro model of PD and attempted to reveal the potential underlying molecular mechanism.

## Results

### ART attenuated MPP^+^-induced cytotoxicity in SH-SY5Y cells

**Fig. 1:**
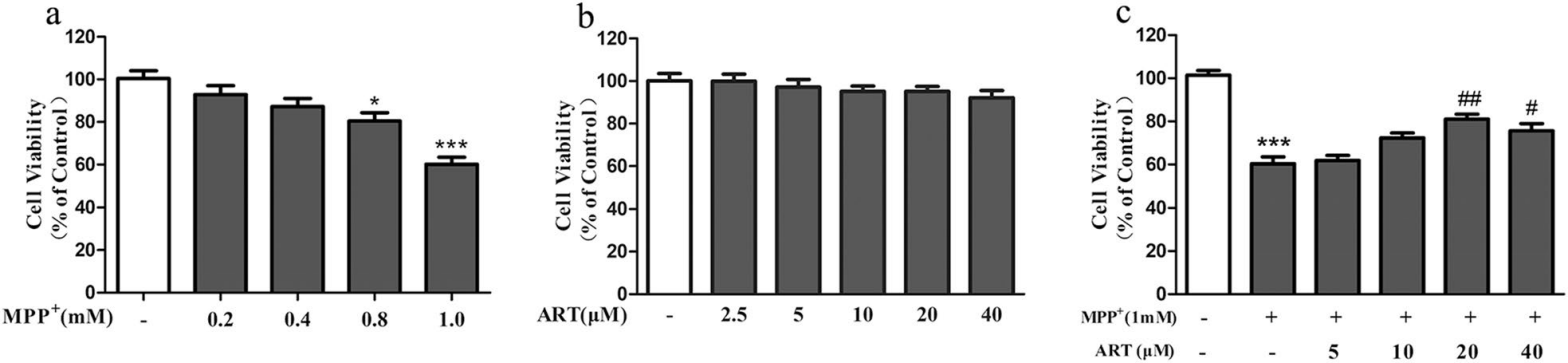
ART reduced the MPP^+^-induced cytotoxicity in SH-SY5Y cells. **a** Effects of different concentrations of MPP^+^ on the viability of SH-SY5Y cells. **b** Effects of different concentrations of ART on the viability of SH-SY5Y cells. **c** Effects of different concentrations of ART on the viability of MPP^+^-treated SH-SY5Y cells. The results are presented as the mean ± SD (n = 5), *p < 0.05, ***p < 0.001 vs. control; ^#^*p* < 0.05, ^##^*p* < 0.01 vs. MPP^+^

We treated cells with different concentrations of MPP^ + ^(0.2, 0.4, 0.8, or 1.0 mM) for 24 h, and the results showed that MPP^+^ could significantly reduce cell viability in a dose-dependent manner (Fig. 1a). Next, we treated cells with different concentrations of ART (2.5, 5, 10, 20, or 40 μM) for 24 h, and the results showed that no obvious cytotoxicity was observed at ART concentrations up to 40 μM (Fig. 1b). Then, we treated cells simultaneously with 1 mM MPP^ + ^and different concentrations of ART (5, 10, 20, or 40 μM) for 24 h, and the results showed that compared with the MPP^ + ^group, the group treated with MPP^ +^ and 20 μM ART exhibited the highest cell viability (Fig. 1c). Therefore, in the following steps, we chose ART at a concentration of 20 μM to study its protective effect on MPP^ +^ -damaged SH-SY5Y cells.

### ART decreased MPP^+^-induced oxidative stress in SH-SY5Y cells

**Fig. 2:**
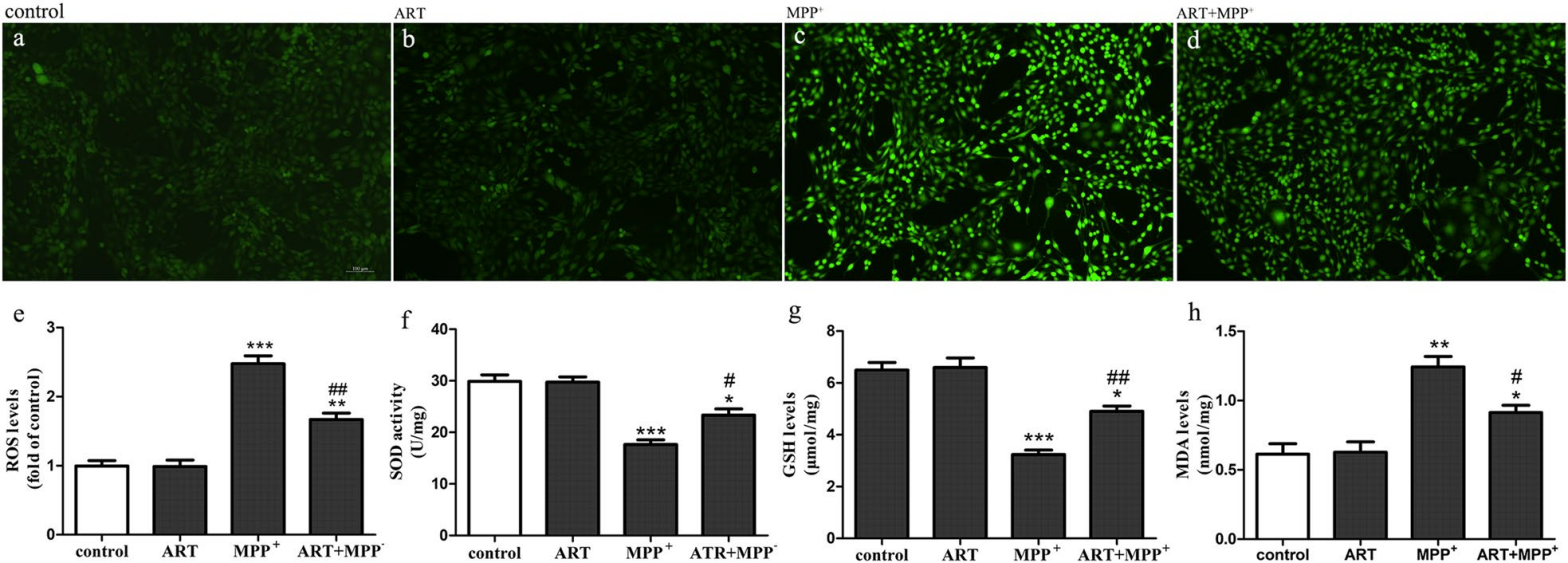
ART reduced the oxidative stress injury induced by MPP^+^. The level of intracellular ROS production was measured using a DCFH-DA fluorescent probe (**a**–**d**), and the fluorescence intensity was analyzed by ImageJ (**e**). The fluorescence intensity of the control group was set to 1, and the fluorescence intensity of the other groups was compared with that of the control group. ART increased the SOD activity (**f**) and GSH production (**g**) and decreased the MDA production (**h**) compared with MPP^+^. Scale bar = 100 μm. The results are presented as the mean ± SD (n = 5), ^*^p < 0.05, ^**^p < 0.01, ^***^p < 0.001 vs. control; ^#^*p* < 0.05, ^##^ *p* < 0.01 vs. MPP^+^

We investigated the effect of ART on MPP^+^-induced oxidative stress injury in SH-SY5Y cells by assessing ROS production, SOD activity, and GSH and MDA levels. DCFH-DA is an indicator of general oxidative stress that was used to measure the level of intracellular ROS production. As shown in Fig. 2a–d, ART significantly reduced the production of intracellular ROS induced by MPP^+^. The fluorescence intensity shown in Fig. 2a–d was analyzed, and the results are shown in Fig. 2e. As shown in Fig. 2e, ROS production increased to 2.48 ± 0.20 after treatment with MPP^+^, which was clearly different from the ROS production in the control cells of 1.00 ± 0.13 (*p* < 0.001, n = 5) (Fig. 2e). However, compared with MPP^+^ treatment alone, simultaneous treatment with ART and MPP^+^ obviously reduced ROS production to 1.67 ± 0.16 (*p* < 0.01, n = 5) (Fig. 2e).

As shown in Fig. 2f and Fig. 2g, compared with the corresponding controls, MPP^+^ clearly decreased the level of SOD from 29.9 ± 2.15 U mg^−1^ to 17.7 ± 1.53 U mg^−1^ and decreased the level of GSH from 6.5 ± 0.5 μmol mg^−1^ to 3.23 ± 0.31 μmol mg^−1^ (*p* < 0.001, n = 5). However, compared with MPP^+^ alone, simultaneous treatment with ART and MPP^+^ clearly increased the level of SOD from 17.7 ± 1.53 U mg^−1^ to 23.3 ± 2.08 U mg^−1^ and increased the level of GSH from 3.23 ± 0.31 μmol mg^−1^ to 4.9 ± 0.36 U mg^−1^.

As shown in Fig. 2h, the content of MDA clearly increased to 1.24 ± 0.13 nmol mg^−1^ in the MPP^+^ group, while this content was only 0.61 ± 0.13 nmol mg^−1^ in the control group (*p* < 0.01, n = 5). Compared with MPP^+^ alone, simultaneous treatment with ART and MPP^+^ significantly reduced the MDA content to 0.91 ± 0.09 nmol mg^−1^ (*p* < 0.05, n = 5).

### ART alleviated MPP^+^-induced Mitochondrial Membrane Potential (MMP) damage in SH-SY5Y cells

**Fig. 3:**
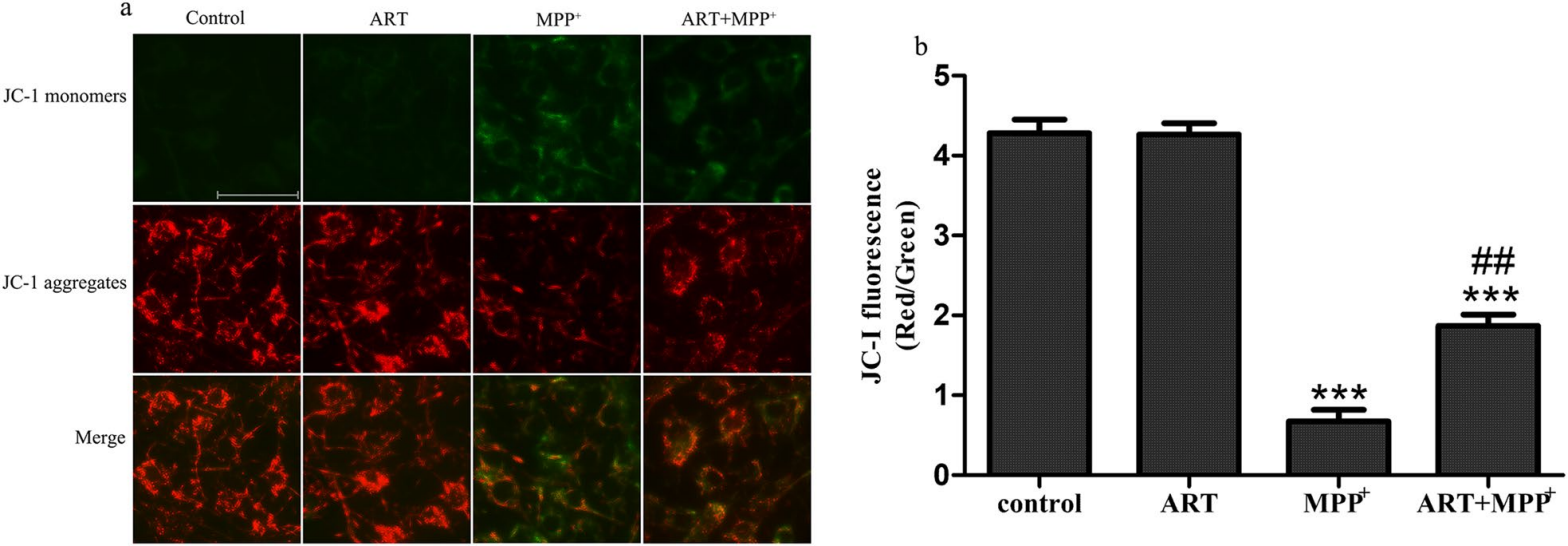
ART alleviated the MPP^+^-induced mitochondrial damage. In the JC-1 staining data, red fluorescence represents JC-1 aggregates and indicates that the MMP is normal, and green fluorescence represents JC-1 monomers and indicates that the MMP is decreased (**a**). The ratio of red fluorescence intensity to green fluorescence intensity was analyzed by ImageJ (**b**). The results are presented as the mean ± SD (n = 5), Scale bar = 50 μm. ^***^p < 0.001 vs. control; ^##^*p* < 0.01 vs. MPP^+^

JC-1 is an ideal fluorescent probe that is widely used to detect the MMP, and the transition of JC-1 from red fluorescence to green fluorescence can be used as an indicator of early apoptosis. As shown in Fig. 3a, in the control group and ART-treated group, JC-1 staining showed weak green fluorescence and bright red fluorescence. In the MPP^+^ treatment group, JC-1 staining showed increased green fluorescence and decreased red fluorescence, which indicated a significant decrease in the MMP. However, simultaneous treatment with ART and MPP^+^ reduced the dissipation of the MMP, resulting in increased red fluorescence and decreased green fluorescence. The ratio of red fluorescence to green fluorescence represents the degree of MMP depolarization (Fig. 3b).

### ART decreased MPP^+^-induced apoptosis in SH-SY5Y cells

**Fig. 4:**
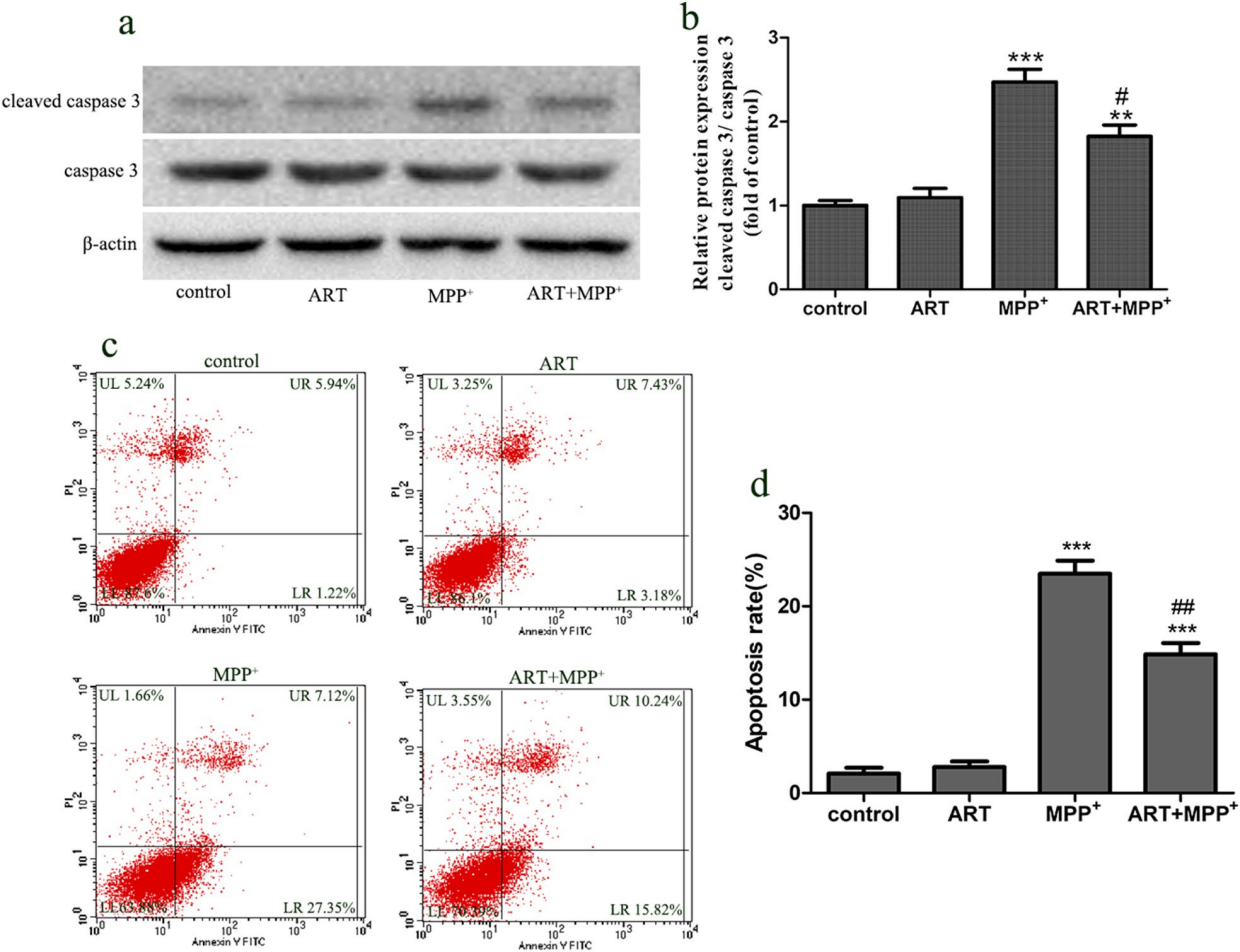
ART reduced the MPP^+^-induced apoptosis in SH-SY5Y cells. The protein expression of caspase-3 and cleaved caspase-3 was detected by Western blotting, and β-actin was used as an equal loading control (**a**, **b**). Flow cytometry was used to detect cell apoptosis after double staining with annexin V-FITC and PI (**c**, **d**). The data are presented as the mean ± SD (n = 3). ^**^ *p* < 0.01, ^***^ *p* < 0.001 vs. control; ^#^*p* < 0.05, ^##^*p* < 0.01 vs. MPP^+^

To study whether ART can reduce the apoptosis of SH-SY5Y cells injured by MPP^+^, we detected the protein expression of caspase-3 and cleaved caspase-3 and performed flow cytometry analysis. The results showed that, compared with the control, MPP^+^ significantly increased the relative expression of cleaved caspase-3. However, compared with MPP^+^, simultaneous treatment with ART and MPP^+^ significantly reduced the protein expression of cleaved caspase-3 (Fig. 4a–b). In addition, we used flow cytometry to detect cell apoptosis. In the control and ART treatment groups, the levels of apoptosis were very low. After MPP^+^ insult, the apoptosis rate increased from 1.22% to 27.35%. However, after simultaneous treatment with ART and MPP^+^, the percentage of apoptotic cells decreased to 15.82% (Fig. 4c–d).

### ART decreased the level of autophagy induced by MPP^+^

**Fig. 5:**
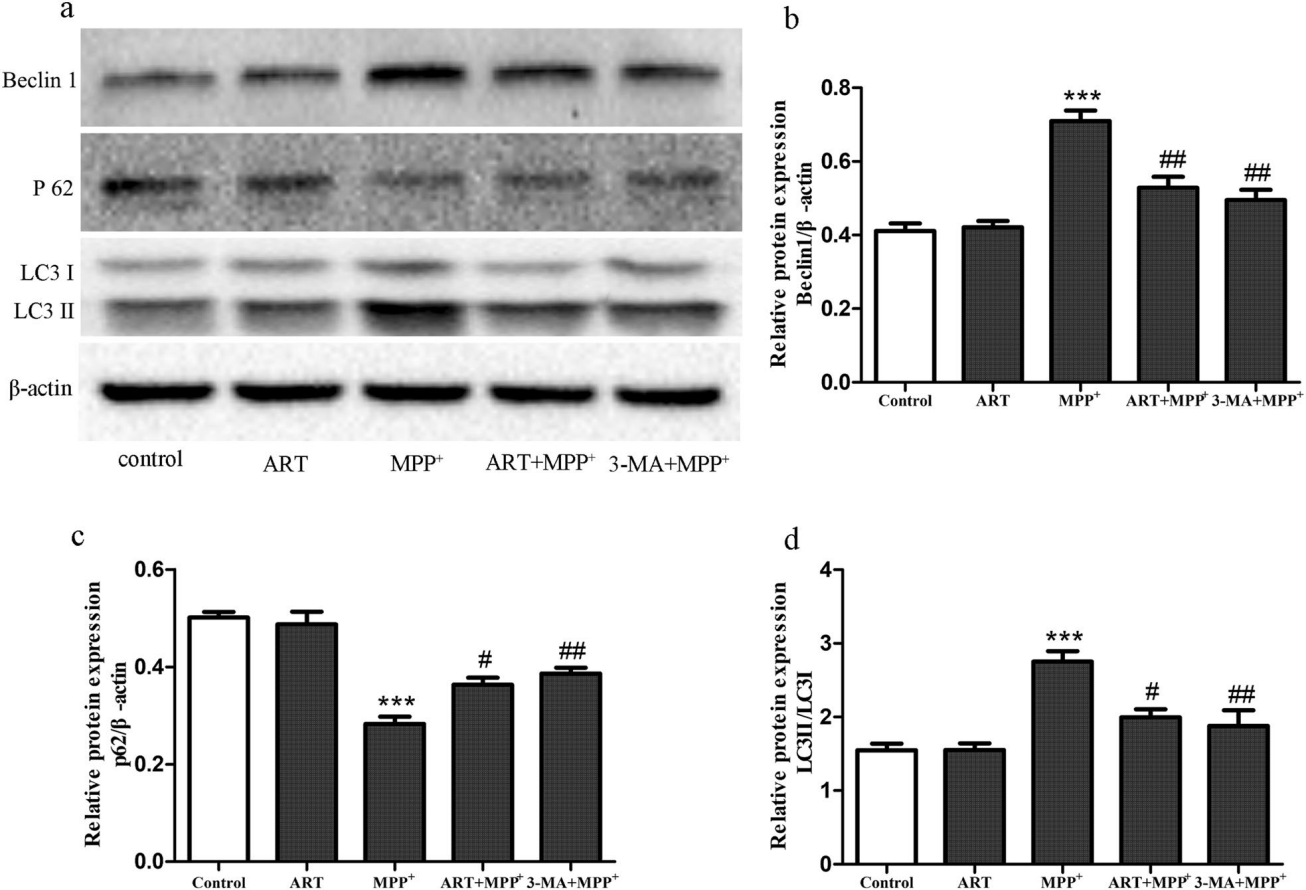
ART inhibited the autophagy induced by MPP^+^ in SH-SY5Y cells. The protein expression of beclin-1, p62 and LC3 was detected by Western blotting, and β-actin was used as an equal loading control (**a**–**d**). The data are presented as the mean ± SD (n = 3). ^***^p < 0.001 vs. control; ^#^*p* < 0.05, ^##^*p* < 0.01 vs. MPP^+^

To study the effect of ART on autophagy in MPP^+^-treated SH-SY5Y cells, we detected the expression of beclin-1, p62 and LC3, which are marker proteins of autophagy. As shown in Fig. 5a–d, compared with the control, MPP^+^ treatment significantly increased the protein expression of beclin-1 and the ratio of LC3II/LC3I but decreased the expression of p62, indicating that MPP^+^ treatment induced autophagy. However, after simultaneous treatment with ART and MPP^+^, the protein expression of beclin-1 and the ratio of LC3II/LC3I decreased, but the expression of p62 significantly increased. Interestingly, ART showed the same effects as 3-MA in regulating autophagy-related protein expression. These results suggested that ART could inhibit the autophagy induced by MPP^+^.

**Fig. 6:**
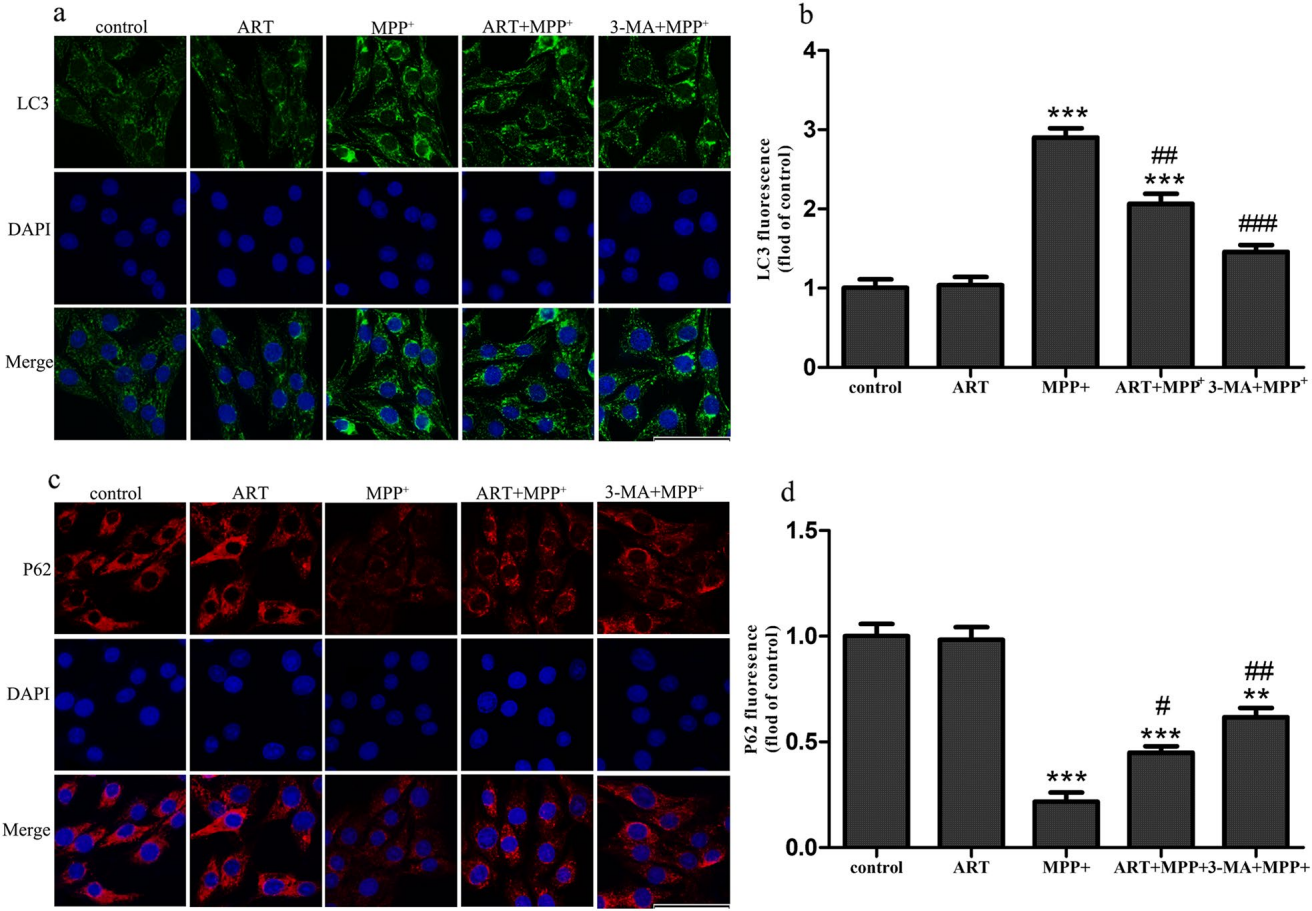
The protein expression of LC3 based on immunofluorescence staining (**a**), and the fluorescence intensity was analyzed by ImageJ (**b**). The protein expression of p62 based on immunofluorescence staining (**c**) and the fluorescence intensity was analyzed by ImageJ (**d**). The fluorescence intensity of the control group was set to 1, and the fluorescence intensity of the other groups was compared with that of the control group. The data are presented as the mean ± SD (n = 3), Scale bar = 50 μm. ^*^p < 0.05, ^**^p < 0.01, ^***^p < 0.001 vs. control; ^#^*p* < 0.05, ^##^*p* < 0.01 vs. MPP^+^

To verify the results described above, we performed immunofluorescence staining. As shown in Fig. 6a–d, compared with the control, MPP^+^ significantly increased the expression of LC3 and decreased the expression of p62. However, compared with MPP^+^ alone, simultaneous treatment with ART and MPP^+^ decreased the expression of LC3 and increased the expression of p62. These results were consistent with the Western blotting results.

## Discussion

MPP^+^, an active metabolic byproduct of MPTP, is a neurotoxin that can be taken up by dopamine transporters into the mitochondria of dopaminergic neurons and inhibit the activity of mitochondrial complex I. The reduction in mitochondrial complex I activity can lead to oxidative stress damage to neuronal cells and ultimately lead to dopaminergic neuron death [15]. That is, MPP^+^ can selectively destroy dopaminergic neurons. In fact, treatment of SH-SY5Y cells with MPP^+^ to induce damage is a common method used to establish PD models in vitro [16].

It is well known that ART is one of the best drugs for treating malaria, and its use has been promoted worldwide [17]. In recent years, studies have found that in addition to its antimalarial effects, ART also has neuroprotective effects [18]. In fact, the antioxidant and neuroprotective effects of ART have been studied in the context of another neurodegenerative disease, Alzheimer’s disease. Sarina et al. [19] reported that ART protects PC12 cells from β-amyloid-induced apoptosis by activating the ERK1/2 signaling pathway. In our study, we found that ART could significantly increase the viability of MPP^+^-damaged SH-SY5Y cells, significantly reduce oxidative stress damage and reduce MMP depolarization. In the apoptosis study, we found that ART reduced the protein expression of cleaved caspase-3 and reduced the rate of cell apoptosis caused by MPP^+^, which were consistent with previous studies. These results indicated that ART is a potential neuroprotective drug that can inhibit the apoptosis of dopamine neurons caused by oxidative stress.

Autophagy is a process by which cells degrade their own organelles and misfolded proteins [20]. A large number of studies have shown that autophagy plays important roles in the occurrence and development of PD (e.g. [21, 22]). However, the specific mechanism by which autophagy functions in PD models is unclear. One study showed that there was a substantial loss of dopaminergic neurons in the substantia nigra and a substantial degree of autophagy in a MPTP-induced model of PD in rhesus monkeys [23]. These results were further proved in C6 cells. Another study showed that MPP^+^ disrupted the autophagic flux in a PC12 cell model, leading to a significant increase in the number of LC3-positive autophagic vesicles [24]. These results showed that the activation of autophagy may be a part of the programmed cell death triggered by neurotoxins [25], which is called "autophagic cell death—ACD". ACD can also be observed in animals and patients with PD [25]. Our study showed that the protein expression of the autophagy markers LC3 and beclin-1 increased in MPP^+^-treated SH-SY5Y cells, while the expression of p62 decreased. This result indicated that MPP^+^ treatment induced the occurrence of autophagy, and this conclusion is the same as that of previous studies [26, 27]. In addition, we found that ART treatment could reverse the increase in the autophagy levels caused by MPP^+^, indicating that the antioxidant effect of ART in MPP^+^-treated SH-SY5Y cells may be related to autophagy. Therefore, we hypothesize that the antioxidant and antiapoptotic effects of ART in MPP^+^-treated SH-SY5Y cells may be related to ACD. However, the specific mechanism of autophagy in PD still requires further research.

At present, the drug treatments for PD are limited to dopaminergic agonists and cholinergic antagonists, but long-term use of these drugs is associated with many adverse reactions. Antioxidants can prevent the neuronal damage caused by oxidative stress. Therefore, identifying high-efficiency, low-cost, low-toxicity and even nontoxic natural antioxidants to prevent or treat neurodegenerative diseases has attracted substantial attention. ART exhibits low toxicity. Clinical studies and meta-analyses showed that no serious side effects were observed during the use of artemisinin, and serious adverse reactions have not been reported after long-term use [28]. To the best of our knowledge, our study is the first to reveal that ART exerts a neuroprotective effect in cell models of PD through its antioxidant activity, but our research still has some limitations. More specifically, (1) our research was conducted under in vitro conditions. Therefore, it is necessary to conduct in vivo experiments in future studies; (2) it showed that autophagy was involved in the mechanism underlying the neuroprotective effect of ART. Due to time and other reasons, we were unable to conduct further studies on this specific mechanism. However, our future research will explore this mechanism.

## Conclusion

In conclusion, our results indicate that ART protects dopaminergic neurons from MPP^+^-induced damage by reducing oxidative stress and apoptosis, and these effects may be related to the inhibition of autophagy. Therefore, our research reveals a new drug with potential protective effects on dopamine neurons in PD, which may provide hope for the prevention and treatment of PD in the future.

## Methods

### Cell culture and drug treatment

The human neuroblastoma cell line SH-SY5Y was obtained from Sun Yat-Sen University (Guangzhou, China) and cultured in DMEM/H medium (HyClone, Logan, UT, USA) with 10% fetal bovine serum (Gibco, Grand Island, NY, USA) and 1% glutamine in a humidified incubator with 5% CO2 at 37°C. We changed the culture medium every two days and subcultured the cells when the density reached 80%. (1) To study the effect of MPP^+^ and/or ART on cell viability, we treated the cells with the indicated concentrations of MPP^+^ and/or ART for 24 h and performed a CCK-8 assay. (2) To study the protective effect of ART on MPP^+^-treated SH-SY5Y cells, we treated the cells with PBS, ART, MPP^+^, or ART + MPP^+^ for 24 h. The concentration of ART was 20 μM, and the concentration of MPP^+^ was 1 mM. (3) To study changes in autophagy, we treated the cells with PBS, ART, MPP^+^, ART + MPP^+^ or 3-MA + MPP^+^ for 24 h. The concentration of ART was 20 μM, the concentration of MPP^+^ was 1 mM, and the concentration of 3-MA was 5 mM. MPP^+^ was purchased from Sigma (Sigma, D048, St Louis, USA). ART was purchased from Nanjing DASF biotechnology  co., Ltd (Nanjing, China). The autophagy inhibitor 3-methyladenine (3-MA) was purchased from Sigma (Sigma, 189490, St Louis, USA).

### Cell viability

Cell viability was measured by CCK-8 assay (Solarbio, CA1210, Beijing, China), according to the manufacturer’s instructions. Briefly, 5 × 10^3^ SH-SY5Y cells per well were plated in 96-well plates and incubated for 24 h. Then, the cells were treated with MPP^+^ and/or ART for another 24 h. Then, 100 μl culture medium containing 10 mM CCK-8 was added to each well and incubated at 37°C for 2 h. The absorbance was measured at 450 nm with a multimode microplate reader (EnSpire, PerkinElmer, Singapore). The cell viability of the control group was set to 100%, and the cell viability of the other groups was compared with that of the control group.

### Intracellular reactive oxygen species (ROS) detection

The levels of intracellular ROS were measured by a ROS assay kit (Beyotime Biotechnology, S0033, Shanghai, China), according to the manufacturer’s instructions. After drug treatment, the SH-SY5Y cells were incubated with serum-free medium containing 10 μM DCFH-DA at 37 °C for 20 min and then washed with serum-free culture medium three times. After adding 1 ml fresh medium to each well, the fluorescence was observed using the GFP channel of an inverted fluorescence microscope (Nikon, ECLIPSE Ti, Tokyo, Japan). The fluorescence intensity was analyzed by ImageJ.

### Superoxide dismutase (SOD) activity assay

The intracellular SOD activity was measured with a total SOD activity detection kit (Beyotime Biotechnology, S0101, Shanghai, China), according to the manufacturer’s instructions. After drug treatment, the cells were washed once with PBS and collected by centrifugation. The cells were fully lysed in SOD sample preparation solution, and the supernatants were collected at 12,000 × *g* at 4°C for 5 min. The absorbance was detected at 450 nm and 600 nm (reference wavelength) with a multimode microplate reader, and the SOD activity was calculated.

### Glutathione (GSH) assay

The content of GSH was measured with a glutathione assay kit (Beyotime Biotechnology, S0052, Shanghai, China), according to the manufacturer’s instructions. After drug treatment, the cells were washed once with PBS and collected by centrifugation. After the samples and standards had been prepared, the absorbance was detected at 412 nm. We generated a standard curve and calculated the contents of GSH in the samples based on this standard curve.

### Malondialdehyde (MDA) assay

The intracellular MDA levels were measured with an MDA detection kit (Beyotime Biotechnology, S0131, Shanghai, China), according to the manufacturer’s instructions. After drug treatment, the cells were collected. The cells were lysed, and the supernatants were collected at 10,000 × *g* for 10 min. After the samples and standards had been prepared, the absorbance was detected at 532 and 450 nm (reference wavelength). The MDA contents in the samples were quantified based on the standard curve.

### Mitochondrial membrane potential (MMP) assay

To monitor the mitochondrial integrity, a mitochondrial membrane potential assay kit with JC-1 (Beyotime Biotechnology, C2006, Shanghai, China) was used, according to the manufacturer’s instructions. Briefly, after drug treatment, the SH-SY5Y cells were incubated with JC-1 working solution at 37°C for 20 min and washed twice with JC-1 buffer. Red fluorescence and green fluorescence were observed with the GFP channel and TRITC channel of an inverted fluorescence microscope (Nikon, ECLIPSE Ti, Japan). The fluorescence intensity was analyzed by ImageJ.

### Flow cytometry assay

We used an Annexin V-FITC cell apoptosis detection kit (Beyotime Biotechnology, C1062M, Shanghai, China) to detect cell apoptosis, according to the manufacturer’s instructions. After drug treatment, the cells were harvested and resuspended in PBS. A total of 50,000 to 100,000 cells were collected and resuspended in 195 μl Annexin V-FITC binding buffer, and then, 5 μl Annexin V-FITC and 10 μl PI were added with gentle mixing. The cells were incubated at room temperature for 20 min, and then, the fluorescence was detected by flow cytometry (BD, Accuri C6, New York, USA).

### Western blot assay

Cell lysates were prepared, and the protein concentrations were quantified with a BCA protein assay kit (CWBIO, CW2011S, Beijing, China). Samples with equal amounts of proteins were separated by 12% SDS-PAGE and transferred to PVDF membranes. After blocking with 5% skim milk at room temperature for 1 h, the membranes were incubated at 4°C overnight with primary antibodies (1:1000) against cleaved caspase-3 (Cell Signaling Technology, Aps175, Boston, USA), caspase-3 (Abcam, ab90437, Cambridge, UK), LC3 (Abcam, ab128025, Cambridge, UK), Beclin1 (BD, 612113, New York, USA), p62 (Abcam, ab56416, Cambridge, UK) or β-actin (1:5000; CWBIO, CW0096M, Beijing, China). The next day, the membranes were washed with TBST three times and incubated with HRP-conjugated anti-rabbit or anti-mouse secondary antibodies (1:5000, CWBIO, Beijing, China) at room temperature for 1 h. The blots were visualized with a cECL Western blot kit (CWBIO, CW0049M, Beijing, China), and the images were analyzed by ImageJ.

### Immunofluorescence staining

SH-SY5Y cells were seeded on slides in 12-well culture plates. After drug treatment, the cells were fixed with ice-cold methanol for 5 min, blocked with 1% bovine serum albumin (BSA) for 30 min, and then incubated with primary antibodies against LC3 (1:200) and P62 (1:100) at 4°C overnight. The next day, the slides were incubated with Alexa Fluor 488 (Bioss Antibodies, bs-0295G-AF488, Beijing, China) or Alexa Fluor 555 (Bioss Antibodies, bs-0296G-AF555, Beijing, China) secondary antibodies (1:500) at 37°C for 1 h and incubated with DAPI (Boster, AR1177, Wuhan, China) for 5 min. Fluorescence was observed with a fluorescence microscope (Olympus, BX53, Tokyo, Japan).

### Statistical analysis

The results are expressed as the mean ± standard deviation (mean ± SD). Comparisons among multiple groups were performed with one-way ANOVA followed by Tukey’s post hoc test, and *p* < 0.05 was considered statistically significant.

## Data Availability

All the data generated or analyzed during this study are included in this published article.

## Abbreviations

PD: Parkinson's disease
ART: Artemisinin
MPP^+^: 1-Methyl-4-phenyliodine iodide
DCFH-DA: 2′,7′-Dichloro-dihydro-fluorescein diacetate
ROS: Reactive oxygen species
SOD: Superoxide dismutase
GSH: Glutathione
MDA: Malondialdehyde
MMP: Mitochondrial membrane potential
DA: Dopamine
H2O2: Hydrogen peroxide
PC12: Rat adrenal pheochromocytoma
MA: 3-Methyladenine
MDA: Malondialdehyde
BSA: Bovine serum albumin
MPTP: 1-Methyl-4-phenyl-1,2,3,6-tetrahydropyridine
ACD: Autophagic cell death
L-DOPA: Levodopa
MPTP: 1-Methyl-4-phenyl-1,2,3,6-tetrahydropyridine
WST-8: 2-(2-Methoxy-4-nitrophenyl)-3-(4-nitrophenyl)-5-(2,4-disulfophenyl)-2H-tetrazolium sodium salt
